# The cycle of distrust in health policy and behavior: Lessons learned from the Negev Bedouin

**DOI:** 10.1371/journal.pone.0237734

**Published:** 2020-08-20

**Authors:** Barak Hermesh, Anat Rosenthal, Nadav Davidovitch

**Affiliations:** Department of Health Systems Management, School of Public Health, Faculty of Health Sciences, Ben-Gurion University of the Negev, Beersheba, Israel; University of Lincoln, UNITED KINGDOM

## Abstract

**Background:**

Over the last decades, health systems worldwide have faced a decline in public trust. For marginalized minority populations, who generally suffer from poverty and political exclusion, the roots of this trend go much deeper, establishing a state of bi-directional *distrust* between them and health institutions. Although studied to a lesser extent compared to trust, distrust does impede health initiatives, such as infectious diseases prevention programs, mostly of so-called Neglected Zoonotic Diseases (NZDs). Where distrust prevails, even trust building actions such as defining rights and obligations, prioritizing “the greater good” and increasing transparency, are prone to failure. In this study, we deepen the understanding of the concept of distrust through a unique case study of Brucellosis, a prevalent bacterial zoonotic disease endemic to disadvantaged Bedouin communities in southern Israel.

**Methods:**

In the years 2015–2019, we qualitatively studied socio-political aspects in a governmental Brucellosis control campaign in southern Israel. We used in-depth interviews with 38 governmental and private health workers, agriculture and nature preservation workers, livestock owners and community leaders. Further, we conducted participant observation in 10 livestock pens and in policymaking meetings, and collected policy and media documents in order to triangulate the results.

**Results:**

We conceptualize three different types of distrust between authorities and marginalized communities—“intention-based distrust”, “values-based distrust” and “circular distrust”—to better explain how distrust originates and reinforces itself, reproducing the endemicity of NZDs. Based on that, we portray a practical framework to reduce distrust in health policies, by reframing local discourses, reshaping disease monitoring schemes from enforcement-based to participation-based, and promoting political inclusion of disadvantaged communities.

**Conclusions:**

The suggested analysis and framework redirect health policy objectives to not only acknowledge, contain and reduce the consequences of distrust, but also to strive for societal justice as a tool for health promotion.

## Introduction–trust, distrust, and health policy

Mutual trust between the public and health systems has long been recognized as integral to the long-term success of policy initiatives. Yet trust cannot be assumed, and trust building should be a fundamental part in planning and program implementation [1]. The view of health systems as socio-political entities, or even tools to achieve social justice, underscores the importance of trust in obtaining equality and equity in health promotion.

Mutual trust benefits both governments and societies; it fosters the legitimacy of government and its ability to influence people’s lives. Moreover, it builds vibrant social communities and helps to foster social cohesion between a government and its citizens [2–5]. However, trust in public institutions, in general, and health systems, in particular, is deteriorating [4–6]. Commonly ascribed social factors to this trend include the rise of individualized autonomy, the decline in deference to authority, and even the breakdown of communities and social networks [7,8]. Others point to economic factors, such as global commercialization and the rise of a neo-liberal economy, which also affect public trust in state institutions, among them the healthcare system. However, building and maintaining trust is crucial to cost containment and public cooperation, especially in universal health systems from which opting out is not an option [8,9].

By contrast, declining trust should not be conflated with *distrust*, for which there is much less theory and policy research. Trust and distrust are not end-points on a single continuum; instead, these are distinct constructs with different determinants and effects on stakeholders [6,10,11]. Using these terms interchangeably negates important differences between the two, and hinders differential analysis of their respective effects on planning and implementation of public health interventions. Indeed, research suggests that distrust significantly affects the effectiveness of health policies, such as interventions to contain infectious diseases [12,13]. Nevertheless, more research and theory is required to understand the factors that determine and sustain distrust, especially its presentation in specific local contexts.

Where distrust exists, trust-building initiatives are insufficient or often ineffectual. To demonstrate, we will first distinguish trust from distrust, and describe how theoretical foundations of trust in health systems are undermined when applied to marginalized populations. We will examine the case of brucellosis, an emerging infectious zoonotic disease endemic to livestock tended by Bedouin communities in the Negev desert of southern Israel. Brucellosis can be transmitted to humans via direct contact with infected livestock or consumption of unpasteurized dairy products; It results in a febrile illness, arthritis and enlargement of the spleen and liver, and it could also lead to multiple chronic complications if left untreated [14].

This analysis provides a frame of reference germane to various marginalized societies in modern states, and the viability of traditional livelihoods, including agro-pastoral livestock rearing in contemporary economies. We identify and describe the cycle of reciprocal reinforcement of distrust of health policy between neglected populations and governments, and assert that *distrust reduction*, rather than traditional trust building strategies, serves as a more effective platform upon which to plan health policy and interventions. Finally, we present a practical framework for health policy planning, which aims to acknowledge, reframe, and break the cycle of distrust.

## Background

### Trust and distrust in disadvantaged populations

*Trust* is built over time based on expectations of how other people and systems will behave in the future [2,15]. Thus, in order to trust, people need to actively and willingly make themselves vulnerable to powerful others. Various authors have proposed differing theories and determinants of trust. For example, exponents of “rational choice” theory view trust as a strategic decision, a self-interested exchange, built upon risk-assessment [2,16]. Others claim that such rationale calculations are uncommon, and suggest an “affective” notion of trust based on emotional bonds developed over repeated interactions [3]. Taking a normative view, some see trust as more altruistic, rooted in belief in the goodwill of others [5]. Whether it be knowledge, emotions, or norms that guide trust or some combination of the three, trust is forged in the context of relationships and complex social systems. Accordingly, health systems operate with the trust of the public, and provide a vital basis for societal exchange, but in order to do so, they must relate to the role of socio-political factors in the public’s willingness to trust and in the system’s trust-based policies towards the public [8,17].

*Distrust*, however, is a distinct construct with distinct effects on stakeholders [11]. While public trust in government is created and sustained through effective communication, stability of social order, accountability, and enactment of rights and obligations [1,18], distrust has different determinants. It is the feeling of betrayal or significant disappointment produced by incongruence of cultural values [6,10]. Suspicion is a core element in distrust, a central cognitive component that entails doubt in the other’s *motives*, rather than their *actions* [4]. It is said, however, that trust can wear out with disuse and neglect, and is difficult to re-construct once broken, potentially leading to distrust [8]. The consequences of trust and distrust are also distinct. While trust reduces transaction costs and increases social capital, distrust inhibits agency, mobilizes defensive attitudes, fosters suspicion of hidden actors with mal-intent, or alternatively leads to “inaction” by both the government and the public [6,18].

Application of health policies that aim to establish trust is especially challenging with disadvantaged subpopulations. To demonstrate, we will present three differing debates. The first debate addresses the notion of trust as a right conditioned upon fulfillment of obligations. O’Neil espouses the idea of trust as the basis for human rights and democracy, rather than rights and democracy being the basis for trust. The basis for trust itself, she claims, is a systematic account of obligations of citizens and public institutions alike; otherwise, a passive notion of rights is assumed and it is unclear “who is required to do what for whom.” [7,19] Nonetheless, other scholars have argued that democratic welfare states should institutionalize social rights, and that lacking, inaccessible, or incomplete fulfilment of these rights is indeed the cause for public’s distrust [18,20]. Similarly, health systems that struggle to meet their obligations due to economic processes such as privatization, serving mostly strong and wealthy populations, further undermine their own trustworthiness [8].

A second debate addresses trust as integral to the success of health initiatives, and thus questions the extent to which governments should *compel* compliance with public health policy. The utilitarian view contends that a certain degree of compulsion is required. In utilitarian societies, trust is built when the public believes that government agencies exercise power judiciously [19]. Often, however, utilitarian policies that are based on lack of trust in citizens, and use compulsion in order to achieve the “greater good,” adversely undermine reciprocal trust that forms the foundation of a legitimate government. When utilitarianism is applied uncritically in public health, individuals, mostly from marginalized populations, are sacrificed for the good of many without any protection of their rights [21].

A third debate touches upon the ability of public institutions to build trust simply by increasing transparency. Actually, trust is important only when conditions of dependence and uncertainty exist in mutual relations [10]. For example, as much as it is crucial for physicians to be trustworthy in the eyes of their less knowledgeable and less powerful patients, it is also important for physicians to trust their patients and rely on their accounts, motives and cooperation [22]. Accordingly, communicating data between public agencies and citizens and vice versa does not necessarily lead to trust; instead, trust is the basis upon which information credibility is built [17]. Information about values and intentions of health institutions, thus, will do more to build trust than data on their performance and competencies shared in the name of transparency [15]. Moreover, transparency is less helpful to the “information poor” who lack the resources to question experts and to make a reflexive choice to trust and, thus, are further disadvantaged and marginalized [17]. Further, these populations are frequently excluded from the creation of epidemiological evidence due to presumed “subjectivity” and “irrelevance” of their local knowledge [23]. This may lead to policies that are considered by policymakers as irrefutably good or bad, based on oversimplification of the data epistemological role and its supposed objectivity, ignoring contexts in which diseases emerge [21].

### Zoonotic diseases–neglect, vulnerability, and distrust

Brucellosis is a *neglected zoonotic disease* (NZD), one of about 200 different infectious diseases that transfer from non-human to human animals and are responsible for 60% of all human infectious diseases [24,25]. Although incidence of NZDs, usually categorized under the larger category of other *emerging infectious diseases*, has risen in recent decades, they receive little attention relative to the extent of their impact. This disregard is greatest with poor ethnic minorities and livestock-rearing communities [26]. Over 600 million people globally are livestock dependent, and represent up to 70% of the world’s rural poor; up to 20% suffer from NZDs and associated consequences [25,27].

This neglect has been blamed on the complexity of NZDs, which are described as “wicked problems.” [28] While clinicians claim that variable symptomatology hinders diagnosis and causes under-estimation and under-investment [26], disease ecologists point towards the multi-factoriality of these diseases. NZDs are caused by a combination of environmental factors such as urbanization, which enables pathogen–vector–host interactions [29,30]; economic factors, such as global trade, which enables disease dissemination [31]; and political factors, such as social inequalities, which affect vulnerable populations most heavily [32]. This array of factors, recently described as “structural drivers of vulnerability to zoonotic diseases,” [33] drives distrusting action–reaction (and also “inaction,” as later explained) in policy and behaviors, hinders reporting of livestock disease [34], fuels vaccination opposition, and hampers bio-security [35,36].

### The case study of brucellosis in southern Israel

Brucellosis is the most common NZD worldwide, with an annual incidence of 0.5M human cases. Referring to the perpetual inattention it receives, being a disease of the poor, as opposed to its heavy annual economic burden, brucellosis is described as “the definition of a neglected disease.” [37,38] It is transferred from livestock, mostly sheep and goats, to humans by contact or proximity to livestock, or by consumption of unpasteurized dairy products infected with the bacteria *Brucella* spp. [39]. Although it increases the risk of miscarriage in livestock, animal carriers appear largely un-symptomatic. In humans, however, brucellosis causes various acute symptoms such as intermittent fever and arthritis, and chronic debilitating complications such as sacroiliitis and endocarditis that can even lead to death [14].

Brucellosis is endemic in most low- and middle-income countries, especially in the Middle East, complicated by illiteracy, population over-distribution, and the under-advancement of health networks [40]. In Israel, brucellosis prevalence varies significantly between ethnic groups and social classes, and is most prevalent among the Bedouin living in the Negev desert of southern Israel, a minority group at the bottom-most socio-economic cluster in Israel that have historically practiced sheep, goats and camels rearing [41]. The prevalence of brucellosis in this community has recently increased, reaching a peak in 2014 at 153.8 cases per 100K people, second in incidence only to Syria [42,43].

Governmental programs to prevent brucellosis in human populations first aim to reduce prevalence in its animal hosts. The main effort is herd vaccination and annual serological testing, as well as culling of infected animals, as no treatment has yet been developed. Long-term disease reduction requires improvement of hygiene practices in high-risk populations and enhanced agricultural bio-security measures including animal monitoring and movement control, and management of biological waste [44]. The multiplicity of tasks, roles, and interests of stakeholders–government agencies, NGOs, and community members–and the multiple interactions derived from them, requires “societal consensus” and trust building among various governmental and municipal authorities, as well as local communities, prior to implementation [45].

In Israel, the institutional response to brucellosis is managed and administrated by the Department of Veterinary Services and Animal Health (IVS), subject to the Ministry of Agriculture and Rural Development; however it is also supplemented by other ministries and NGOs. Due to several bureaucratic, political and budgetary constraints, the last two campaigns aiming to eradicate brucellosis were both terminated prematurely, before reaching a satisfactory reduction in the disease prevalence [43,46].

Such constraints, common also to various neglected zoonoses other than Brucellosis, were addressed since the beginning of the 21^st^ century by the One Health approach [47,48]. As an inter-disciplinary approach to health problems affected by the interconnections between animals, humans and natural resources, One Health has globally promoted collaborations in research and policy making, and resource pooling of veterinary, public health and environment protection institutions [49]. The focus of One Health on health professions’ inter-disciplinarity, however, has been criticized for its reductionism, due to the fact that more complex socio-political factors that produce health disparities and distrust in health systems have been often left unaddressed [28,50]. In this article, we suggest a step-back from simple inter-disciplinarily and professional collaboration, and a move towards a political and historical exploration of the essence of government and minority population relations that constitute the settings for disease prevention.

### Histories of the Bedouin and Israel–marginalization and livestock rearing

The Negev Bedouin are a Muslim Palestinian minority who comprise 3.5% of the population of the State of Israel [51]. The term Bedouin (from Arabic: “desert dwellers,”) which also describes other populations in the Middle East and Africa, indicates a certain descent characterized by tribal social structure and nomadic-pastoral livelihood practices. Over the 20^th^ century, these features have been greatly affected by economic and political powers of nation-states that forced the Bedouin into permanent settlements and modern labor markets [52].

However, disagreements over locations and livelihood resources, which characterize most post-nomadic and modern-state interrelations, are overshadowed in the Negev desert case by the “Israeli-Palestinian conflict” [53]. After the establishment of Israel and the 1948 War, most Bedouin left or were expelled, while those who remained were geographically concentrated far from the Israeli–Egyptian border for security reasons and placed under military rule for two decades [52,54]. Meanwhile, Israel claimed ownership over most Negev desert lands previously cultivated by Bedouin, deeming their inhabitants as “illegal intruders” [55], while strengthening Jewish holding of the lands [56]. Today about a third of the Bedouin still live in 35 settlements that are unrecognized by the state and, thus, deemed “illegal.” Located on state lands, these villages comprise of mostly tents and cinder-block shacks, are deprived of services, and lack any official status [57]. The rest of the Bedouin population in the Negev desert lives in government-planned urbanized Bedouin towns, that are at the bottom-most cluster of the socio-economic scale, with deficient roads, water, and electricity infrastructure [58]. These towns receive poor educational, health, and employment services [59], leading to lower health and social outcomes compared to other Israeli towns [58,60]. Moreover, while the population’s birth rates are among the highest in the country [60], the government’s failure to provide adequate housing motivates illegal construction. The consequential governmental demolition of an increasing number of dwellings and agricultural structures each year [61,62] inflicts terror, severe trauma, and major depression on Bedouin families, and is perceived as a “manifestation of discrimination, oppression, and exclusion,” reinforcing the circle of distrust [63–65]. Recent governmental efforts have been made to “regularize” Bedouin settlements; however, they all clash with the longstanding conflict between the population and the authorities over land rights, cultural preservation, and autonomy [66].

Historically, nomadic pastoralism was the main source of income for the Bedouin, but in recent decades, most men have entered the workforce as blue-collar laborers [67]. However, many families continue to rear livestock, and while some have developed high-yield commercial systems, many households rear small, unprofitable subsistence herds to maintain familial cohesion, social networks, and, ultimately, their Bedouin identity [68]. Currently, about 2,000 Bedouin herders rear about 400,000 sheep and goats (most of the livestock in Israel). These figures are low estimates due to a lack of data for many unregistered herds [69]. The proximity of people to livestock in Bedouin towns, as well as poor hygiene practices, are important risk factors for human infection in NZDs, especially brucellosis, as demonstrated in many other post-nomadic populations worldwide that are also undergoing urbanization [70,71].

Examining distrust, using the case of brucellosis interventions must be understood in context of these various historical and contemporary public–government relations, and how they affect livelihoods and human–animal interfaces. This framing does not only explain the context for intentions-based, values-based, and circular distrust, but further shapes distrust as a construct that requires attention in public health policy. The study of distrust, thus, is inherently contextual and can benefit from an analysis based on the perspectives of stakeholders themselves [1].

## Methods

This study was conducted between 2015 and 2019 in order to map various stakeholders who deal with brucellosis in the Negev desert, and analyze historical and political aspects of their perceptions of interventions. It was approved by the Ethics committee of the Faculty of Health Sciences of Ben-Gurion University of the Negev. Data collection was carried out before, during, and after the Israeli government's intervention to reduce the disease prevalence in the Negev desert following its steady increase in the human population, which was led by the Veterinary Services of the Ministry of Agriculture and Rural Development between 2015 and 2017.

We interviewed 38 stakeholders in brucellosis policy planning, health provision, and livestock rearing, following receiving written from each one of the interviewees. Interviews were conducted with twelve Ministry of Agriculture employees and municipal and private veterinarians; eleven Ministry of Health and Health Maintenance Organization employees as well as physicians working in the Negev desert; eleven livestock owners and NGO workers; and four private educators, nature preservation workers, and entrepreneurs. The interviewees were selected based on principles of achieving “maximum variation” of disciplines and priorities and highlighting “critical cases” of policymaking and field experience [72]. The interviews were in Hebrew and in Arabic conducted using a semi-structured interview guide, asking mainly open-ended questions in order to focus on the interviewees’ perceptions of brucellosis, its causes, implications on health and society, and intersections across and within institutions. The interviews lasted one to three hours and were recorded with participants’ consent.

Participant observation was conducted in four livestock pens that varied in their degree of agricultural productivity, between extensive pasture to intensive production, in order to examine distinct human–animal interfaces that affected livelihood and disease transmission. These observations lasted for about half an hour each. Participant observation was conducted during six meetings with policymakers such as inter-ministerial meetings, government round-tables, and parliamentary assemblies. Each of these observation lasted for the duration of the meetings, usually several hours of each meeting.

Data from the interviews and observations are not publicly available for anonymity and privacy reasons. Ethics approval was obtained from the Faculty of Health Sciences, Ben Gurion, University of the Negev IRB. A request can be sent to: ethics@medic.bgu.ac.il.

Table 1 summarizes details of the data collection from these interviews and observations:

**Table 1:** Summary of data collected from interviews and observations.

Study method	Discipline/sector	Institute/social group	Number of interviewees / observations
Interviews	Herder community	Livestock owners/herders	7
		NGO representatives	4
	Animal health providers	Israeli Veterinarian Services workers	3
		Ministry of Agriculture workers	4
		Municipal veterinarians	2
		Private veterinarians	3
	Human health providers	Ministry of Health workers	5
		Health Maintenance Organization (HMO) workers	2
		Physicians who work in the Negev	4
	Miscellaneous	Educators, nature preservation workers, entrepreneurs	4
Total interviews			38
Observations	Policymaking meeting	Round-table conferences	2
		Inter-ministerial meetings	2
		Parliamentary assemblies	2
	Human–animal interface	Sheep and goat pens	3
		Camel pen	1
Total observations			10

Lastly, policy documents, reports by the State Comptroller and relevant NGOs, and media reports were collected via Google and searches of government websites (e.g., Ministries of Agriculture and Health). We collected various types of data from a range of sources to enable triangulation and to increase the breadth and integrity of the data until reaching saturation.

All data extraction, indexing, and analysis was done by the first author, a Jewish Israeli MD–PhD candidate, studying sociological approaches to health policy. When necessary, a Palestinian research assistant provided Arabic–Hebrew translation during and following the interviews. An advantage of data collection by neither a veterinarian nor Bedouin was the justification for numerous inquiries about day-to-day encounters, practices, and perceptions. The study was supervised by the other authors, a medical anthropologist and a public-health researcher.

Several ethical considerations were taken into account following qualitative inquiry guidelines [73]. First, informed consent was obtained by acquainting the participants with the study’s researchers, methods and goals, allowing them to decide whether to participate or not, as well as to be recorded or not. Second, anonymity and confidentiality were kept by aiming to discuss institutional roles and cooperation activities, rather than specific colleagues’ opinions or behaviors. Following interviews, sorting and indexing of all raw recorded and transcribed data was done only by the first author, and the analysis and writing process was done with deidentified data. Privacy was specifically important in this study, in which relative small number of participants, whether policy makers, care providers or livestock owners participate in close inter-personal collaborations. Third, in order to protect participants from harm, considering the fundamental power inequalities between different stakeholders, some politically sensitive issues were introduced at the opening of the conversations, clarifying to participants their right to refuse to answer any question, and guaranteeing their confidentiality.

The analysis process included primary coding of all transcribed text, the formation of hierarchies between the codes, a focused analysis of significant codes and the establishment and clarification of the final categories that emerged from the text. Formation of the category of distrust and its sub-categories followed Shkedi’s principles of constructivist qualitative thematic analysis, combining inductive ground-up conceptualization, based on the coded text segments, with a deductive theoretical framework, based on the literature of trust, in order to build upon contemporary theoretical discourses [74]. This analysis was conducted using Atlas.ti versions 7 and 8.

## Results–classification of distrust

Distrust between the public and the government emerged as a main theme in analyses of political and historical perceptions of brucellosis and its intervention in southern Israel. We identified three distinct forms of distrust applicable to both the government and the public: *Intention-based distrust* stemming from concerns regarding the malevolent actions of others who aim to harm; *Values-based distrust*, which is harder to validate as it is a deeper, more embedded form of distrust built upon perceived incongruence of identities and values; and *Circular distrust*, which entails the consequences of distrust expressed through explanations of cause and effect, used by interviewees to explain their distrust of behavior or policy, shielding them from the dangers of the other’s deeds. As its name suggests, it is difficult to identify the locus of circular distrust as it is sustained bi-directionally; thus, it is also hard to oppose or negotiate. Circular distrust can sometimes lead to *inaction* that perpetuates neglect of zoonotic diseases as well as negative inter-relations in general.

### Intention-based distrust

Intention-based distrust in grounded in the belief of others’ malicious intentions. This distrust is not the uncertainty of others’ ability to perform their tasks or obligations according to rules, rather it is a notion based on personal or collective experience of the others’ wish to harm. In this respect, most prominent was a common belief among Bedouin livestock keepers that the Israeli government’s hidden goal is to eliminate Bedouin livestock. Explaining the substandard governmental attention to brucellosis in the Negev desert, a livestock owner who holds about 500 hundred goats, sheep, and camels on the outskirts of a government-planned Bedouin town claimed:

*The Ministry of Agriculture* (*MoA*) s*hould care for us*, *but would they shed a tear if our flocks die*? *This government is against the Bedouin*. *They want to eliminate the flocks*. *Why*? *I suspect to drive the Bedouin out of the desert and into the towns*.

A Bedouin MoA employee further elaborated on this point. In the following quote, he refers to “The Green Patrol”, a governmental unit for the supervision of open spaces, subjected to the Israel Nature and Parks Authority, and to the “Pitzuah”, a MoA unit which is entitled to expose, prevent, and enforce agricultural violations:

*Most herders believe that the government wishes to eliminate the sheep*. *There is some truth to that*, *based on the past activity of the Green Patrol*, *the law against”the black goat*.*”*–Do people still remember this?–*Yes*! *And the fines they get from the Pitzuah while grazing*, *and the pens’ demolitions*…

Similarly, several studies found that policies restricting grazing lands, promoting sedentarization and income-taxing livestock trade are perceived as government efforts to rid the Negev desert of the herds, and consequently of the Bedouin traditional way of life altogether [75,76]. This belief has both historical roots and contemporary support. Historically, forced sedentarization of the Bedouin comprised multiple state actions. In addition to the aforementioned efforts to geographically concentrate the population, the state applied specific regulations (e.g. the “black goat” law, forbidding over-grazing due to presumed ecological damage), as well as units limiting population movement (e.g. the “Green Patrol” and the “Pitzuah” unit) [76,77]. Enlisting these units in the brucellosis reduction campaign in order to locate unregistered herds [78] may have further undermined trust. As explained by a district veterinarian:

*The Bedouin*, *in his nature*, *is suspicious*. *“Why do you bring in the Government”*? *So I asked* (*the head of the IVS*) *not to bring in the PITZUAH*. *The PITZUAH are enforcement*, *and we don’t want that*.

From the government’s perspective, however, Bedouin herders intentionally undermine policy implementation allowing outbreaks to spread and persist. As was described by a MoH veterinarian who served as the contact person for IVS veterinarians:

*When MoA veterinarians come to cull animals* [infected with brucellosis], *the Bedouin conceal them*, *transfer them* [to other owners], *or claim they were stolen*. *Can you fight it*? *It’s hard*. *It’s a mentality*.

While officials ascribe this behavior to a certain Bedouin “mentality,” a Bedouin (MoH) employee who investigates human brucellosis outbreaks attributes it to inadequate government planning:

*It was not possible in our inquiries to confirm the source of contamination as the* [patient’s] *parents would not cooperate or tell the truth …*. *Such fear*, *suspicion*, *and deliberately withholding information are caused by ambiguous or conflicting policies and unmet promises*, *responsible for a complete loss of trust in the establishment*.

Regardless of whether it is embedded in collective perceptions or individual experience, attributed to the state’s control over ethnic minorities, or to post-nomadic societies’ defiance of regulations, our study shows that *intention-based distrust* influences provision and acceptance of health promotion interventions. These perceptions, as explained by our interviewees, originated in events that followed, and even preceded, the establishment of the Jewish State of Israel, as far as the colonial history of the Middle East. Ignoring or attempting to reshape this long-standing culture of distrust seems not only to reproduce a demonizing or generalizing rhetoric between the community and health providers, but also to prevent parties from taking the necessary steps to manage a communicable disease that is well-controlled in most industrialized countries.

### Values-based distrust

Distrust does not only stem from negative perceptions of the other’s intentions. A more insidious kind derives from perceived incongruence in values between a government and recipients of its services. This distrust can lead to even worse consequences, clouding the perception of neutral or helpful inter-personal encounters with stigmatization and generalization. A Bedouin physician who provides education to prevent brucellosis transmission to humans said:

*You can educate people, but this will only change things in future*. *Among the Bedouin, there is indifference—“It will not happen to me*.” *Sometimes, you tell a herder to boil the milk, and he’ll reply “my sheep are fine, I just checked them,” but after a while, he and his children get sick*. *He doesn’t take you seriously. “My father and my grandfather never boiled, and they were just fine*.”

The physician’s claim that education has limited effects on risk perception and health behavior is supported by the literature, and is also demonstrated in a recent Israeli study that found no association between brucellosis knowledge and the actual consumption of non-regulated dairy products [79]. However, when educators attribute this lack of correlation to a general tendency of communities coping with illnesses to disregard their own health, they themselves foster, and eventually reproduce, values-based distrust. This stigmatization characterizes not only health provision encounters, but also those that aim to economically support livestock holders. As claimed by a governmental livestock-rearing guide:

*What I’ve learned over years of working with the Bedouin is that most of what they say is false… Maybe I’m stigmatizing, but I doubt everything they say*. *For example, we attempted to establish a model farm with one herder who agreed that he would track and document his lambs, but when I returned a month later, he had sold them with no documentation*… *There is a credibility problem, but there’s something more; it may be due to suspicion*.

This inability to find common ground may derive from distinct economic worldviews, in which modern agriculture as a business runs up against the view of livestock as a survival safety net for many Bedouin [57]. We contend that this incongruence of worldviews lead to values-based distrust, especially when neglected or explained superficially, creating stigmatizing generalizations and iterative distrust. Interestingly, even a private Bedouin veterinarian had had similar experiences. However, his explanations for this form of distrust were different:

*Governmental offices never included the Bedouin*. *Over time*, *since the British Mandate*, *the Bedouin got it into their DNA*, *that whomever comes from the government is here to ruin your life*. *I have a white Isuzu truck*, *same as the ones used by the* [Israel Land] *Authority*. *Such a vehicle is a threat*, *no matter who the driver*. *I came to take* [blood] *samples from a few camels*, *and this dumb herder photographs my car’s front and back*. *Even though I’m not from the government*, *still they suspect me*.

While the aforementioned encounter is indubitably influenced by distrust, this distrust is attributed here to the continuing exclusion of the Bedouin population in policymaking. Consequently, this exclusion, symbolized by the “white truck,” creates *generalized distrust* in Bedouin communities, impeding their cooperation even with seemingly “neutral” non-governmental health providers. However, this distrust is also attributed to a so-called hereditary characteristic that negates “rational” thinking, conveying a perception of mutual values-based distrust. Referring to a different symbol of ongoing contention between the Bedouin and the government, a livestock owner gave other reasons not to cooperate:

*If someone were to ask me today if I would join a* [governmentally planned] *town*, *I would say no*. *If I wanted to return with my sheep from the pasture*, *I would have nowhere to put them*. *Do they expect me to drop them inside my house*? *But at the same time*, *we see that Jews can obtain Loner Farms—the Bedouin cannot*. *How can you ignore peoples’ culture*, *something they have known all their lives; you can’t change people overnight*. *Then you build a museum to show “how Bedouins used to live*,*” but this is my life*!

This herder’s frustration with the government stems not only from inadequate planning for joint animal–human living, but also from a perceived process of *deculturation* depriving the Bedouin of their historical livelihoods. The museum referred to by the herd owner was designed and curated by Jewish Israelis, and was criticized as being structured for the pleasure of western tourists: “There is an implicit presumption that Bedouin material culture and activities represented are no longer viable or extant today, but serve as ‘artefacts’ from a culture that once was” [80]. Moreover, this herder feels discrimination due to the recent establishment of “Loner Farms (havot bodedim),” a state project that has granted several Jewish families ownership over vast Negev desert lands. In addition to this project being critiqued for having jeopardized distributive justice and multi-ethnic planning [81], its perceived partiality acts, in this case, as the main motivation for distrust.

In these contexts, the *Bedouin museum* that aims to preserve cultural history represents domination, similar to how the *white truck* that provides accessible health services represents exclusion. Ultimately, the contradictory meanings contained in these symbols is based not only on values incongruence, but also on incongruent power relations, societal status, and life realities.

### Circular distrust

Circular distrust results when stakeholders distrust one another and act accordingly to minimize their short-term risks, but consequently harm others’ efforts. Thereupon, the offended side acts similarly, confirming preconceptions and creating a self-reinforcing bi-directional distrust. Distrusting behaviors, similar to distrusting policies, are perceived by their executers as obliged under the given conditions. As explained by a Bedouin herder:

*When discussing farmers’ difficulties and distrust in the system, the decision to close the livestock markets in Bedouin towns was made* “*from above” on the pretense that infected herds spread brucellosis. Herders just want to make a living, and now they can’t sell their goods. This is collective punishment! If the goal, however, was to stop the trade, it was not achieved*. *People just do it now in various “crooked” ways*.

Where live-animal markets exist, brucellosis control is more challenging for they increase local epizootic and zoonotic transmission of diseases [82]. The decision “from above” to eliminate this means of survival subsistence, however, may have encouraged herders to trade illegally, in “crooked” ways—a presumed cause for the spread of brucellosis outside the Negev desert to northern Israel [41]. This risk-reduction decision, so it seems, excluded, and thus exacerbated, the factor of government–public distrust. Trust was similarly noted as a reason for herders’ resistance to the culling of their brucellosis-infected animals. A representative from a herders’ NGO explained:

*The MoA must earn the herders’ trust*. *Otherwise*, *it is impossible to eradicate the disease*. *If you* [as the government] *would say*, *“your sheep is infected*, *here is ₪500*,*” I would hand over a different sheep worth₪ 500 [or less]*, *and sell the infected sheep for ₪1*,*200 by taking the ear-tag out of this one and putting it in the other*. *Then what have you done to the disease*? *You gave me the power to transmit it to others*.

Circumventing the system by exchanging infected sheep, which adversely affects brucellosis control efforts, is justified here by distrust caused by unmet expectations for compensation. However, according to a MoA manager, the compensation was already set at the highest possible rate for exactly the same reasons:

*In the 1990s*, *we were paying too much*, *and herders* intentionally *infected their animals to receive compensation*. *Let’s say a herder has a flock worth little in the market*. *If the animals become infected*, *he will get a much higher price from the government; he hits the jackpot*.

While government officials adopt distrusted policies and exclude the public from policy design, as happened with the setting of the compensation policy, they will likely face consequential distrusting behavior, justifying their policy and their decision to limit stakeholder input post factum. Applying a “rational choice” prism here, either to understand what incentivizes the public, or what is included in policy risk-assessment, downplays the inherent distrust factors and the "burden of history" of ongoing relationships that enlist contradictory argumentations (e.g. paying too much leads to deception versus paying too little leads to deception) to decision making. In the case of brucellosis management, distrusting policy further excluded the public, not only from planning, but also from implementing the program. As noted by a private veterinarian who tried to obtain brucellosis test results taken from a flock tended by a herder he serves:

*I asked the* [MoA worker], *“This herder is my client*, *I need to know his test results*.*” He answered that 37 out of 168 of his sheep are infected*, *“but I can’t tell you which ones*… *If the herder knows*, *he’ll sell them*.*” And when herders ask about the results*, *they are told “We’ll let you know when we come to cull your herd*.*” Some wait up to seven months; meanwhile*, *more sheep become infected*.

As concluded by a recent State Comptroller report, extended wait times, limited resources, and existing compensation policies enable brucellosis transmission in the Negev desert [46]. Withholding information from herders during those wait times acts as both a source and consequence of circular distrust. In this manner, distrust is a form of *inaction*, a dangerous byproduct carried out by both the affected public and authorities. This explains why, despite health risks, those who distrust the authorities may conceal health information, hide infected animals, and ignore public health directives. Concomitantly, the Israeli government had done little over decades to manage brucellosis until peak incidence in 2014. Even then, intervention was minimal, failing to cull infected animals, which would have required financial compensation of herders according to the Animal Diseases Directive, 1985 [83]. One policy maker explained the reasons for the refusal:

*You can*, *in fact*, *compensate for culled animals under the Animal Diseases Directive*, *but only if IVS requirements are met*, *and the Bedouin never meet these criteria*. *This means that they cannot be paid under the directive*.*–*Did you try to find herders who do meet the criteria?–*There just aren’t any*. *You have”Father Abraham” strolling around on the hills*. *Do you expect him to invite the IVS to vaccinate the herd*?

The assertion that *all* Bedouin herders would not qualify for the directive’s compensation was inevitable, according to the MoA, who framed it as “the unique social reality” in the Negev desert [84]. However, this form of “social sensitivity,” because of which a decision was made to allocate unique ad-hoc funds to compensate herders without them needing to apply any criteria, actually resulted in delay in animal culling by several years–until those funds were finally earmarked. Moreover, these funds were not legislated and could have been discontinued at any point, as indeed happen with the last two brucellosis campaigns. Consequently, objectives were not achieved, and brucellosis transmission remains a public health problem. To this day, culling and compensation under the Animal Disease Directive is carried out only when brucellosis is detected in commercial barns [85], yet culling and compensation for the Bedouin themselves has been halted.

As described, distrust can take various forms, stem from attributions of malign intentions or contradictory worldviews, build on historical or contemporary issues, and result in benign neglect or direct harm. Distrust is not simply a lack of trust, and should be tackled differently; otherwise, it becomes self-fulfilling, justifying generalizations, stigmatizations, exclusion, and noncooperation. Nor do we call for disregarding the harm of distrust and blindly adopt trust, or to simply “trust trust and distrust distrust” [86] because distrusting behaviors and policies do jeopardize human, financial, social, and cultural resources. We contend that new approaches are necessary. As policies, much like behaviors, are justified by “risk-management,” they can also stem from, and cause, deep distrust. But how can we break this cycle?

## Discussion–why is distrust circular, and how to break the cycle?

Distrust is a multi-faceted concept that characterizes long-standing interrelations. Where it exists, distrust passively reduces interactions, neglecting hazards that continue to spread, or actively sabotages necessary collaborations by means of exclusion and deception. To the detriment of public health, distrust between health systems and underserved populations reinforces preconceptions of value-incongruence and belief in the malicious intent of others. The study of brucellosis in the Negev desert of Israel demonstrates how distrust is circular, self-fulfilling, and multi-directional. It is grounded in history and socio-political factors that affect communities, human–animal interfaces, and government–public relations that cannot be rectified overnight. Later in this discussion, we portray a stepwise framework of distrust reduction to guide health policy interventions. First, the framework explains how decisions that are superficially attributed to governmental risk analysis or to the public’s mentality and culture stem from distrust. Then we suggest approaches to redirect planning towards trustful relations.

The case of brucellosis allows us to demonstrate how common trust promotion mechanisms, such as clear definitions of rights and obligations, utilitarian approaches, and data transparency are destined to fail when distrust prevails. Within this cycle of reciprocal distrust, the principle of transparency that supports stakeholder participation coexists alongside the perceived need to conceal information from distrusted populations. The motivation to allocate ad-hoc resources towards health promotion coexists with halting of more sustainable resources, reducing governmental accountability, and thus destabilizing the balance of rights and obligations. The desire to enforce “the greater good” coexists with dismissal of the interests and rights of minorities. Such conflicting mechanisms lead policymakers to adopt certain “go-to” solutions, such as the under-compensation for animal diseases to prevent coincidental hitchhiking, adversely reinforce existing distrust.

Nevertheless, how can distrust be justified or rationalized in light of the negative long-term consequences? Game theory suggests that individuals who usually obey social rules, will disobey if they cannot trust others to do so as well, even if it is irrational from the systems’ perspective [87]. Another common justification is that when distrust is maintained and reinforced, interactions between communities, and the trust they require, become more problematic and costly [11]. The problem is, again, the vicious cycle of distrust. In health systems, for example, individuals who feel discrimination from health providers seek healthcare less often even though they suffer worse health outcomes, which leads to further deterioration of health that further increases the costs for health institutions [88].

**Fig 1:**
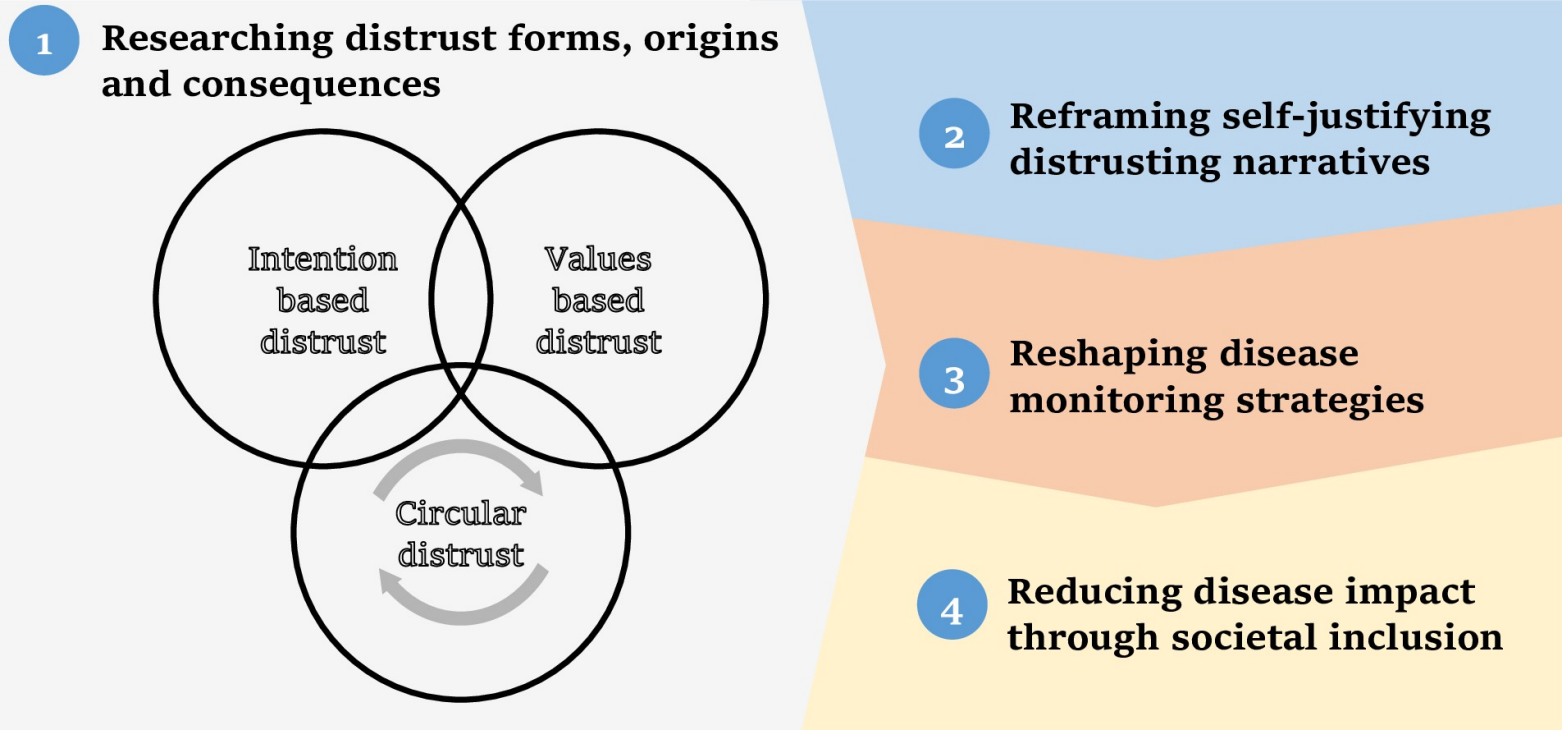
The four-step framework to decrease the burden of distrust in order to tackle health issues in under-served populations.

**Fig 2:**
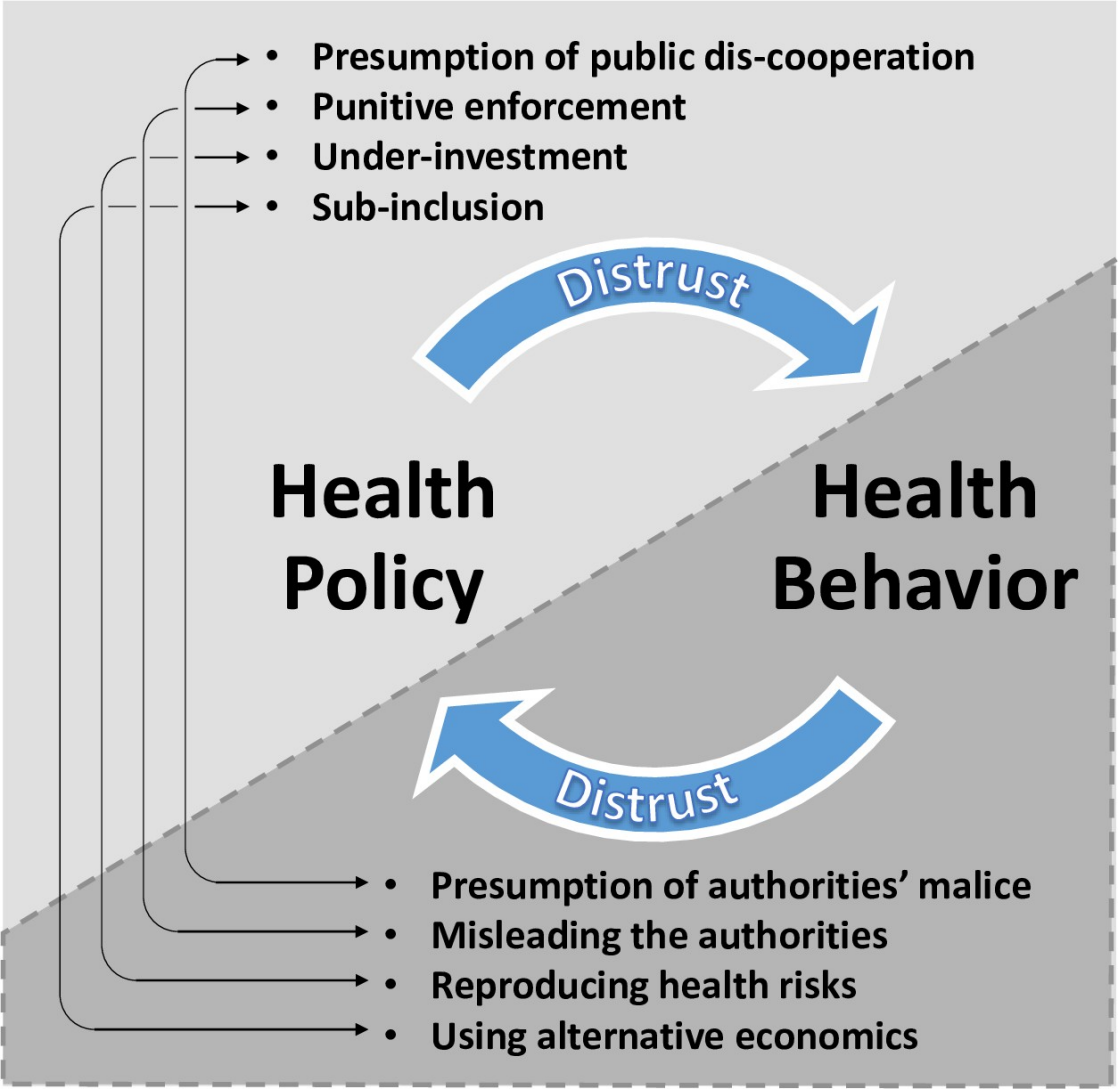
Graphic description of circular distrust between health systems and disadvantaged communities.

What can be done, then, to break the cycle of distrust? We would like to suggest a practical four-step framework, illustrated as follows (Fig 1):

First, existing circular distrust necessitates thorough exploration of its historical, social, and economic factors and consequences, done with diverse key stakeholders and shared with policymakers. It is important to notice and clarify not only distrust based on perceptions of malice intentions, but also distrust that stems from perceived value-incongruences, dividing societies and attributing conflicting meanings to their symbols. Even in developed countries, incongruence between the values of health systems and the public has created generalized distrust in all government initiatives [9]. The suggested preliminary process of distrust exploration can raise much opposition, but should be referred to as a crucial foundation in the reconstruction of health goals and policies. To simplify, this research product could be illustrated in a diagram of factors that reinforce circular distrust, such as follows (Fig 2), broadly applicable to contexts of intervention in disadvantaged populations:

Second, it should be understood that not much can be done to shape traditions of trust built upon historical experiences that societies have undergone [18]. These traditions not only shape the way policy is constructed, but also the rhetoric by which communities cope with diseases, often demonizing public health workers [89]. However, using insights produced in the first step, the self-justifying discourse around distrust could and should be changed. Worldwide, marginalized societies suffer from poverty and denial of autonomy, while at the same time, they are expected to adhere to social norms. Exclusion from the national social sphere encourages reliance on illegal, informal markets and survival by illegitimate means, underserved, but also under-protected, by the law [90]. This framing, we believe, can be more accurate and constructive than the view of poor individuals as criminals or “hitchhikers” that are oblivious to their own best interests, as the view of government ministries intentionally striving to eliminate cultures and peoples.

Third, punitive efforts to enforce compliance with health policy should be reduced as much as possible. In addition to costs associated with human resource requirements, enforcement responds effectively only to *reliability* issues that underlie trust violations, however ineffectively to *value-incongruence*s that underpin distrust [10]. To reduce this kind of distrust, enforcement should be infrequent—a distant protective framework for spontaneous trustful actions, signaling to the public that breaches of trust are rare [18]. Compared to enforcement, approaches to infectious disease monitoring that are built on participation, such as Participatory Epidemiology and Community-Based Monitoring are used worldwide in low-resource settings in a more successful manner. In addition to providing databases for monitoring, they constitute cross-sectoral collaborations in health promotion [91,92].

Fourth, strategies that parallel enforcement reduction must be participatory and inclusive; the need for coercive mechanisms is infrequent when communities can influence their realities by legitimate means. Inclusion only begins with inviting marginalized populations to set their own goals, to develop strategies, and to determine incentives, as well as implement the interventions. More broadly, inclusion goes beyond the traditional parameters of health provision, and adopts holistic approaches that transcend discipline boundaries, knowledge types, and class. Elsewhere, we suggests the “political One-Health” approach to answer such needs [93]. In this case, promoting infrastructure and services for Bedouin settlements, as well as supporting traditional livestock rearing as a sustainable mean of subsistence are investments that are in the public interest beyond the need to eliminate zoonoses. Despite multiple challenges in the contextual political atmosphere, attaining these goals would not only foster the well-being of those most in need, but also begin the process of reducing distrust.

## Conclusions

This study adds new perspectives to the concept of distrust between marginalized communities and health systems. It builds on the claim that trust and distrust are not opposites, but distinct conditions of interrelations that entail distinct approaches. Unlike previous works that focused on trust in order to explain distrust, we dissemble and reconstruct the concept of distrust through the exploration of a unique case study of Bedouin communities and health providers in the Negev desert in the south of Israel, dealing with the neglected zoonosis of Brucellosis. Our analysis highlights and explains three interrelated features of distrust based on: perceived intentions to harm, rooted in history of domination and exclusion; incongruent values such as attitudes towards livelihoods, fostering stigmatization, and victim blaming; and finally, the circulatory aspect of distrust that uses short-term risk-reducing policies or loss-aversion risk behaviors to reinforce distrust and self-confirm its preconceptions.

This study on brucellosis in the Negev desert was conducted within a specific time and location. Therefore, data collection and sampling were dependent, among other things, on personal traits or social networks of the authors, availability of resources, and mere fortuity, thus, anchoring the results in a very specific context. Furthermore, this article focused in distrust between local populations who suffer from diseases and health institutions as a whole. Due to considerations of analysis depth versus expansion, this article does not address distrust within these two sub-groups themselves, which might also influence intervention in neglected zoonosis–such as intra-institutional power relations [50], or gender inequalities that play a substantial role in traditional societies. This factors, however, are nonetheless important and are addressed in other publications resulting from this work.

Nevertheless, using the resulting categorization, we demonstrated how theoretical pillars of trust-building policies such as (top-down) definitions of rights and obligations, enforcement of “the greater good” (while sacrificing minorities), and transparency of (some but not all) information causes only the deepening of distrust and further marginalization of marginalized populations. Consequently, we offer a methodical four-step framework to reduce distrust by exploring and mapping its sources, acknowledging and changing its discourses, and reducing the influences of distrusting actions, first through a decrease in governmental compulsion, and then through inclusion of considerations that go beyond disease elimination and promote social justice. This framework, we believe, can provide tools for the enhancement of acceptance and long-term success of health initiations. More broadly, it can establish healthier relationships between governments and minority populations in the presence of the contemporary trust decline towards health systems specifically, and democratic regimes in general.
